# “I Cannot See”; Inverse Anton’s Syndrome: A Case Study

**DOI:** 10.1192/bjo.2025.10713

**Published:** 2025-06-20

**Authors:** Obumneme Chinweuba, Peter Knynenburg

**Affiliations:** Kent and Medway NHS Social Care Partnership Trust, Maidstone, United Kingdom

## Abstract

**Aims:** Abnormalities of vision have long been documented in psychosis. One syndrome of interest is Inverse Anton’s syndrome. This is a rare manifestation of visual abnormality where a person describes being blind despite objective evidence against this. In this case report, we discuss a patient who presents with a complaint of blindness despite evidence to the contrary.

**Methods:** Case Report.

A 46-year-old male part-time worker, with a childhood history of photosensitive myoclonic seizures which were treated with anti-epileptic medications. At 21 years, he required mental health services as he complained of episodic blindness. He was diagnosed with a delusional disorder. His symptoms resolved drastically after he was treated on olanzapine which he discontinued and remained well for over 2 decades.

He returned to services following a recurrence. His reported blindness is associated with emotional distress and self-harming. He is able to independently mobilise, complete forms and questionnaires but would insist that he is blind despite doing these. He reported mood changes which were treated with sertraline but with no benefits. He was unsuccessfully treated on olanzapine and then switched to quetiapine.

Cognitive Behavioural Therapy based Initial Interventions were unsuccessfully attempted. He was reviewed by neuropsychiatry and complex psychosis service. He is engaged to Occupational Therapy interventions aimed at maximising practical skills. He was assessed by the ophthalmologist and opticians with no significant findings. He was referred to the neurologist. He had a brain Magnetic Resonance Imaging scan which found no abnormality.

**Results:** Discussion.

Inverse Anton’s syndrome is scantily described. There are few case reports with similar presentations. In a 2019 case report, the presentation was similar to this. The exact cause of this syndrome is unclear. It is thought to result from a structural disconnection of the parietal lobe attentional systems from visual perception. In the absence of radiological evidence, this leads to a suggestion of a functional illness or a neuropsychological syndrome in which visual perception and cognitive awareness are dissociated. In the absence of known secondary gain, it continues to present a diagnostic dilemma. This puzzle requires multidisciplinary efforts to solve. Management is focussed on secondary prevention and rehabilitation to improve quality of life.

**Conclusion:** Inverse Anton’s syndrome continues to present with unclear aetiology and diagnostic dilemma. It is hoped that multidisciplinary efforts together with advances in neuroimaging would help understand this syndrome better. We hope that our case report contributes to the body of knowledge and adds more perspective to this.

